# The association of IL-17A polymorphisms with IL-17A serum levels and risk of ischemic stroke

**DOI:** 10.18632/oncotarget.21498

**Published:** 2017-10-05

**Authors:** Hua-Tuo Huang, Yu-Lan Lu, Rong Wang, Hai-Mei Qin, Chun-Fang Wang, Jun-Li Wang, Yang Xiang, Jing Guo, Yan Lan, Ye-Sheng Wei

**Affiliations:** ^1^ Department of Clinical Laboratory, Affiliated Hospital of Youjiang Medical University for Nationalities, Baise 533000, Guangxi, China; ^2^ Youjiang Medical University for Nationalities, Baise 533000, Guangxi, China; ^3^ Department of Dermatology, The Affiliated Hospital of Youjiang Medical University for Nationalities, Baise 533000, Guangxi, China

**Keywords:** IL-17A, genetic, promoter, polymorphisms, ischemic stroke

## Abstract

The aim of our study was to investigate the association of interleukin-17A (IL-17A) polymorphisms with IL-17A serum levels and risk of ischemic stroke (IS) in a Chinese population. 392 IS patients and 443 controls were included in this study. The polymorphisms of IL-17A gene were determined by Snapshot SNP genotyping assay and DNA sequencing. Serum IL-17A levels were measured by enzyme-linked immunosorbent assay (ELISA). We found that the G allele, GA and GG genotypes, and GA/GG vs. AA model of rs2275913 polymorphism were associated with increased risk of IS even after adjusted by clinical characters such as age, gender and diabetes (G vs. A: OR=1.27, 95% CI, 1.05∼1.54, *P*=0.014; GA vs. AA: OR=1.72, 95% CI, 1.05∼2.81, *P*=0.032; GG vs. AA: OR=1.99, 95% CI, 1.08∼3.67, *P*=0.028; GA/GG vs. AA: OR=1.78, 95% CI, 1.11∼2.86, *P*=0.017). Serum IL-17A levels were increased in IS patients compared with controls (*P*<0.01). Individuals carrying rs2275913 GA or GG genotype present higher serum IL-17A levels compared with the rs2275913AA genotype in the IS group (*P*<0.01). In conclusion, this is the first study reporting the rs2275913 polymorphism as a risk factor for IS, which may be partly explained by influencing the levels of IL-17A cytokine.

## INTRODUCTION

Stroke is a major cause of death and a common cause of long-term disability in the world [1–3]. In China, stroke has turned up to be a heavy burden of family and society, with about 2.5 million new cases and 1.6 million death each year [4]. Ischemic stroke (IS) is the most common type of stroke, accounting for more than 80% of all stroke [5]. Until now, the exact etiology of IS has not been fully elucidated. Several non-genetic factors such as age, gender, hypertension, diabetes mellitus, smoking, alcohol abuse and hypercholesterolemia have been demonstrated to play crucial role in the pathogenesis of IS. Moreover, data from family and twins researches have revealed that genetic factors may also play a role in the pathogenesis of IS [6–8].

Interleukin-17A (IL-17A), a member of the IL-17 cytokine family (IL-17A to IL-17F), has been demonstrated to paly crucial role in the progress of atherosclerosis [9–16]. The expression of IL-17A was increased in patients with coronary artery disease (CAD) and IS [12, 17, 18]. High concentration of IL-17A in atherosclerotic plaques can promote the development of atherosclerotic lesions [15], while blockade of IL-17A in atherosclerosis mice model resulted in a significant reduction of atherosclerotic lesions [9, 16]. Additionally, by binding to the heterodimers receptor, IL-17RA-IL-17RC, on different cell types, IL-17A can stimulate the secretion of a series of pro-inflammatory cytokines, such as IL-6, tumor necrosis factor-α (TNF-α), and IL-1β [19–21], and leukocyte-mobilizing cytokines, such as the chemokine (C-X-C motif) ligand 1 (CXCL1), and CXCL8 [22–24]. These cytokines have been previously reported to play a role in the pathogenesis of IS [17, 25–31]. Taken together, these findings indicate that IL-17A may be used as a biomarker and/or therapeutic target for IS.

IL-17A gene, which is located on chromosome 6p12.23 in human genome, encodes the IL-17A cytokine. Recently, several polymorphisms in the IL-17A gene were identified, among them, rs1974226 and rs3748067 polymorphisms in the 3’ untranslated region (3’ UTR) of the IL-17A gene, and rs2275913 polymorphism in the promoter of the IL-17A gene, which were reported to be functional in influencing individual’s susceptibility to a huge numbers of human diseases, such as gastric cancer [32–35], pulmonary tuberculosis [36], asthma [37–39], osteoarthritis [40], rheumatoid arthritis [41], and CAD [42–44]. However, to date, no study has been reported on the association between polymorphisms in the IL-17A gene and IS risk. Thus, in our present study, we investigated the possible association between three polymorphisms of the IL-17A gene and risk of IS in a Chinese population. Moreover, serum IL-17A levels among IS patients and the control group, and the relationship between these three IL-17A polymorphisms and IL-17A serum levels were also explored.

## RESULTS

### Clinical characteristics of the study population

The clinical characteristics of the IS group and the control group are summarized in Table 1. There were no significantly different between IS patients and the control group with regard to age and gender (*P* = 0.329 and *P* = 0.443, respectively). Diabetes and hypertension were more prevalent in IS patients than those of the control group (all *P* < 0.001). Comparing with the control group, the IS group had significantly higher TG, LDL-C and TCH levels (all *P* < 0.05), but lower HDL-C levels ( *P*< 0.001).

**Table 1:** Clinical characteristics of the IS patients and the control group

Characteristics	Controls (n = 443)	Cases (n = 392)	*P* value
Age, (M ± SD, year)	60.7 ± 10.3	61.8 ± 10.1	0.329
Gender, (%)			
Male	278 (62.8)	256 (65.3)	
Female	165 (37.2)	136 (34.7)	0.443
Diabetes, (%)			
Yes	46 (10.4)	74 (18.9)	
No	397 (89.6)	318 (81.1)	<0.001
Hypertension, (%)			
Yes	112 (25.3)	249 (63.5)	
No	331 (74.7)	143 (36.5)	<0.001
TG, (mmol/L)	1.07 ± 0.42	1.70 ± 1.24	<0.001
LDL-C, (mmol/L)	2.49 ± 0.50	3.02 ± 0.76	<0.001
HDL-C, (mmol/L)	1.74 ± 0.37	1.28 ± 0.33	<0.001
TCH, (mmol/L)	4.83 ± 0.69	5.07 ± 0.93	0.024

M ± SD, mean ± standard deviation; TG, triglycerides; LDL-C, low-density lipoprotein cholesterol; HDL-C, high-density lipoprotein cholesterol; TCH, total cholesterol.

### Association between IL-17A polymorphisms and risk of ischemic stroke

**Figure 1:**
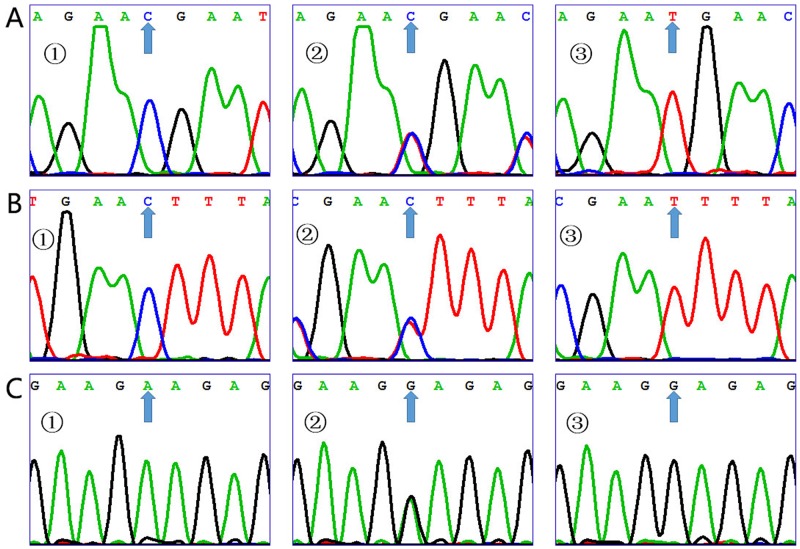
Sequencing map for the IL-17A gene **(A)** Sequencing map for rs1974226. The arrows of figure ①, ② and ③ show CC, CT and TT genotypes for rs1974226 respectively. **(B)** Sequencing map for rs3748067, and The arrows of figure ①, ② and ③ show CC, CT and TT genotypes for rs3748067 respectively. **(C)** Sequencing map for rs2275913. The arrows of figure ①, ② and ③ show AA, GA and GG genotypes for rs2275913 respectively.

All the three polymorphisms showed three genotypes according to the sequencing results (Figure 1). The genotypes distribution of the three polymorphisms in the control group conformed to HWE (Table 2). As is shown in the Table 2, we found that the frequency of rs2275913 G allele in the IS group (52.2%) was significantly higher than those of the control group (46.2%) and was associated with significant increased risk of IS compared with the control group (OR = 1.27, 95% CI, 1.05∼1.54, *P* = 0.014). Table 3 shows the association between IL-17A polymorphisms and risk of IS under genotypes and genetics models analysis. We found that the rs2275913 GA and GG genotypes were associated with significant increased risks of IS compared with the rs2275913 AA genotype between cases and controls, even after adjusted by age, gender, hypertension, diabetes mellitus, TG, LDL-C, HDL-C, and TCH (GA vs. AA: adjusted OR = 1.72, 95% CI, 1.05∼2.81, *P* = 0.032; GG vs. AA: adjusted OR = 1.99, 95% CI, 1.08∼3.67, *P* = 0.028). Moreover, the significant increased risk was also observed in model analysis, even after adjusted by age, gender, hypertension, diabetes, TG, LDL-C, HDL-C, and TCH (GA/GG vs. AA: adjusted OR = 1.78, 95% CI, 1.11∼2.86, *P* = 0.017).

**Table 2:** Allele distributions of the IL-17A polymorphisms between the IS patients and the control group

Polymorphisms	Controls (%)	Cases (%)	OR (95% CI)	*P* value	HWE
rs1974226					0.71
C	836 (94.4)	749 (95.5)	1.00 (Ref)		
T	50 (5.6)	35 (4.5)	0.78 (0.52∼1.22)	0.274	
rs3748067					0.33
C	805 (90.9)	711 (90.7)	1.00 (Ref)		
T	81 (9.1)	73 (9.3)	1.02 (0.73∼1.42)	0.905	
rs2275913					0.16
A	477 (53.8)	375 (47.8)	1.00 (Ref)		
G	409 (46.2)	409 (52.2)	1.27 (1.05∼1.54)	0.014	

OR, odds ratio; CI, confidence interval; Ref, reference category; HWE, Hardy-Weinberg equilibrium.

**Table 3:** Genotype distributions of the IL-17A polymorphisms between the IS patients and the control group

Polymorphisms	Controls	Cases	Logistic regression	
	n=443 (%)	n=392 (%)	OR (95% CI)^†^	*P* value^†^
rs1974226				
CC	394 (88.9)	359 (91.6)	1.00 (Ref)	
CT	48 (10.9)	31 (7.9)	0.84 (0.40∼1.76)	0.643
TT	1 (0.2)	2 (0.5)	2.11 (0.14∼32.99)	0.595
CT/TT vs. CC			0.89 (0.43∼1.82)	0.744
CC/CT vs. TT			2.27 (0.21∼25.09)	0.505
rs3748067				
CC	364 (82.2)	323 (82.4)	1.00 (Ref)	
CT	77 (17.4)	65 (16.6)	1.02 (0.59∼1.77)	0.951
TT	2 (0.4)	4 (1.0)	1.49 (0.15∼14.81)	0.735
CT/TT vs. CC			1.04 (0.60∼1.78)	0.898
CC/CT vs. TT			1.48 (0.15∼14.74)	0.736
rs2275913				
AA	121 (27.3)	72 (18.4)	1.00 (Ref)	
GA	235 (53.1)	231 (58.9)	1.72 (1.05∼2.81)	0.032
GG	87 (19.6)	89 (22.7)	1.99 (1.08∼3.67)	0.028
GA/GG vs. AA			1.78 (1.11∼2.86)	0.017
AA/GA vs. GG			1.36 (0.82∼2.25)	0.229

OR, odds ratio; CI, confidence interval; Ref, reference category; ^†^, Adjusted by age, gender, hypertension, diabetes mellitus, TG, LDL-C, HDL-C, and TCH.

### Genotype distribution of rs2275913 polymorphism in different population

In consideration of the important of the rs2275913 polymorphism in the pathogenesis of IS, we further perform a comparison which shows the genotypes distribution of the rs2275913 polymorphism in different populations (Table 4). The results showed that the genotypes distribution of rs2275913 polymorphism in our current study were significantly different from HapMap-JPT (HM-JPT), HM-LWK, HM-MEX, HM-GIH, HM-YRI, HM-ASW, HM-MKK HM-CEU and HM-TSI populations (*P* < 0.05). However, no significant different was found when comparing with HM-HCB, HM-CHB and HM-CHD populations (*P* > 0.05), (The data of HapMap population were obtained from the website bellow: https://www.ncbi.nlm.nih.gov/projects/SNP/snp_ref.cgi?rs=2275913).

**Table 4:** Genotype distributions of the IL-17A rs2275913 polymorphism in different populations

Population	n	Genotypes (%)			MAF (%)		Ethnic
		AA	GA	GG	G	A	
Present data	443	121 (27.3)	235 (53.1)	87 (19.6)	409 (46.2)	-	Guangxi
HM-HCB	86	14 (16.3)	52 (60.5)	20 (23.2)	-	80 (46.5)	Asian
HM-CHB	82	26 (31.7)	38 (46.3)	18 (22.0)	74 (45.1)	-	Asian
HM-CHD	170	54 (31.8)	78 (45.9)	38 (22.3)	154 (45.3)	-	Asian
HM -JPT^Δ^	172	36 (20.9)	84 (48.9)	52 (30.2)	-	156 (45.3)	Asian
HM-LWK^Δ^	180	-	22 (12.2)	158 (87.8)	-	22 (6.1)	Asian
HM-MEX^Δ^	100	2 (2.0)	52 (52.0)	46 (46.0)	-	56 (28.0)	Asian
HM-GIH^Δ^	176	36 (20.4)	70 (39.8)	70 (39.8)	-	142 (40.3)	Asian
HM-YRI^Δ^	226	-	30 (13.3)	196 (86.7)	-	30 (6.6)	African
HM-ASW^Δ^	98	-	18 (18.4)	80 (81.6)	-	18 (9.2)	African
HM-MKK^Δ^	286	2 (0.7)	28 (9.8)	256 (89.5)	-	32 (5.6)	African
HM-CEU^Δ^	226	28 (12.4)	104 (46.0)	94 (41.6)	-	160 (35.4)	European
HM-TSI^Δ^	176	28 (15.9)	78 (44.3)	70 (39.8)	-	134 (38.1)	European

^Δ^, Comparing with our present data, *P* < 0.05; MAF, minor allele; HM, HapMap; CEU, Utah residents with northern and western European ancestry; HCB, Han Chinese in Beijing, China; CHB, Han Chinese in Beijing, China; CHD, Chinese in Metropolitan Denver, Colorado; JPT, Japanese in Tokyo, Japan; LWK, Luhya in Webuye, Kenya; MEX, Mexican ancestry in Los Angeles, California; GIH, Gujarati Indians in Houston, Texas; YRI, Yoruba in Ibadan, Nigeria. ASW, African ancestry in Southwest USA; MKK, Maasai in Kinyawa, Kenya; TSI, Toscans in Italy.

### Haplotype analysis of rs1974226, rs3748067 and rs2275913 polymorphisms and risk of ischemic stroke

Haplotype analysis was performed and the possible 7 haplotypes are listed in Table 5. We found that the rs1974226 polymorphism was in linkage disequilibrium (LD) with the rs3748067 (D’ = 0.89) and the rs2275913 (D’ = 0.34) polymorphisms. While, the rs3748067 polymorphism was in LD with the rs2275913 (D’ = 0.72) polymorphism. CCA and CCG are the two major haplotypes in both cases (47.0% and 39.4%, respectively) and controls (49.6% and 35.9%, respectively). However, we didn’t find significant association between IL-17A haplotypes and IS risk.

**Table 5:** Haplotype analysis of the 3 polymorphisms and risk of ischemic stroke

Haplotypes	Controls (%)	Cases (%)	OR (95% CI)	*P* value
CCA	439 (49.6)	368 (47.0)	0.84 (0.69∼1.02)	0.075
CCG	318 (35.9)	309 (39.4)	1.10 (0.90∼1.34)	0.350
CTG	58 (6.5)	66 (8.4)	1.26 (0.88∼1.82)	0.208
TCG	33 (3.7)	34 (4.3)	1.13 (0.70∼1.85)	0.614
TTA	2 (0.2)	1 (0.1)	-	-
CTA	6 (0.8)	21 (2.4)	-	-
TCA	15 (1.7)	-	-	-

OR, odds ratio; CI, confidence interval.

### Association of rs2275913 polymorphism and serum IL-17A levels

**Figure 2:**
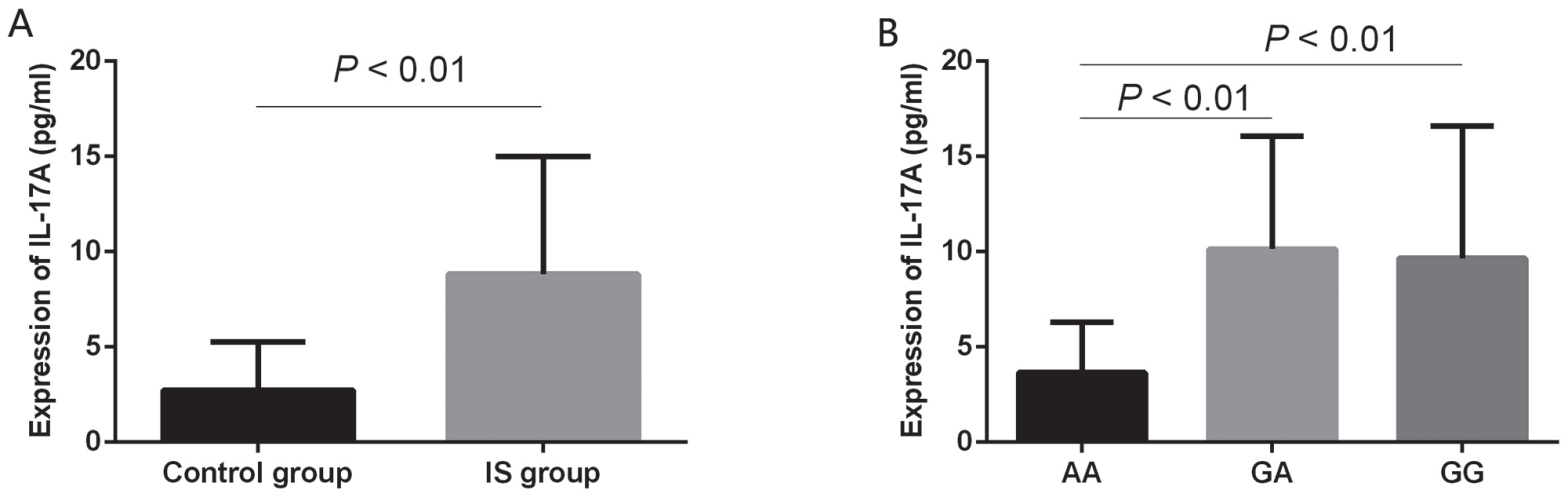
ELISA detection of serum IL-17A levels **(A)** Serum IL-17A levels in the IS group (n = 75) were significant higher than those of the control group (n = 85) (*P* < 0.01). **(B)** Patients carrying the rs2275913 GA (n = 44) or rs2275913 GG (n = 17) genotype present with a higher serum IL-17A levels compared with the rs2275913 AA (n = 14) genotype (*P* < 0.01). However, there were no significantly different in serum IL-17A levels between individuals with rs2275913 GA and rs2275913 GG genotypes (*P* > 0.05). Data are presented as mean ± standard error.

Moreover, serum IL-17A levels among cases and controls, and the association between rs2275913 polymorphism and serum IL-17A levels were examined (Figure 2). We found that serum IL-17A levels in the IS group were significantly higher than those of the control group (*P* < 0.01). In particular, after a systematic comparison between rs2275913 genotypes and serum IL-17A levels, we found that patients carrying the rs2275913 GA or GG genotype present with a higher serum IL-17A levels compared with those carrying the rs2275913 AA genotype (both *P* < 0.01). However, there were no significantly different in serum IL-17A levels between individuals with rs2275913 GA and rs2275913 GG genotypes (*P* > 0.05).

## DISCUSSION

To our knowledge, this is the first research to assess the association between rs1974226, rs3748067 and rs2275913 polymorphisms and risk of IS. The results of our research suggested that the rs2275913 G allele, the rs2275913 GA and GG genotypes, and the GA/GG vs. AA model were associated with significant increased risk of IS. More importantly, we found that the rs2275913 polymorphism was associated with abnormal IL-17A expression. Patients carrying the rs2275913 GA or GG genotype present with a higher serum IL17A levels compared with the rs2275913 AA genotype. Taken together, these results indicate that the rs2275913 polymorphism may serve as a risk factor for predicting IS risk.

The rs2275913 polymorphism is located in the promoter region of the IL-17A gene. Recently, several researches have been conducted to assess the possible association between rs2275913 polymorphism and the susceptibility to cardiovascular diseases, controversial results, however, were obtained, even in the same ethnic group. Geng et al. [42] reported that the rs2275913 AA genotype and the GG/GA vs. AA model were associated with an increased risk of coronary artery disease (CAD) in the Chinese population. However, Shuang et al. [43] reported that the rs2275913 AA genotype and the GA/AA vs. GG model, but no the GG/GA vs. AA model were associated with an increased risk of CAD in the Chinese population. On the contrary, two other Chinese researchers [44, 45] and one Mexico researcher [46] have recently reported that the rs2275913 polymorphism was not associated with risk of CAD. Until now, no study has been conducted to access the association between rs2275913 polymorphism and risk of IS. However, in this study, we found that the G allele, GA and GG genotypes, and the GA/GG vs. AA model of the rs2275913 polymorphism were associated with a 1.27, 1.72, 1.99 and 1.78-fold increased risk of IS, respectively. Moreover, we further investigated whether the rs2275913 polymorphism will affect protein expression of the IL-17A gene. We observed that patients with rs2275913 GA or GG genotype present with higher serum IL-17A levels. The results we obtained is similar to a recent case-control study conducted by Li et al. [47], in which they found that the rs2275913 GG genotype was associated with significantly increased IL-17A levels. In brief, these findings suggest that the rs2275913 GA and GG genotypes are risk factors for IS, probably through upregulation the protein expression of IL-17A gene.

With regard to the rs3748067 polymorphism and risk of cardiovascular diseases, controversial results were also observed. Zheng et al. [44] reported that the rs3748067 CC genotype and the CT/CC vs. TT model of the rs3748067 polymorphism were associated with increased risk of CAD. However, data from case-control studies of Su et al. [45] and Shuang et al. [43] suggested that there were no significantly association between rs3748067 polymorphism and CAD risk. Our present date in agree with the negative result, we didn’t find any association between rs3748067 polymorphism and IS risk.

To date, although association studies between rs1974226 polymorphism and risk of cardiovascular diseases have not been carried out, three independent studies have been conducted to investigate the relationship between rs1974226 polymorphism and risk of human diseases. Korytina et al. [48] reported that rs1974226 polymorphism was associated with a 2.31-fold increased risk of chronic obstructive pulmonary disease under CT/CC vs. TT model in a Russia population. Zhou et al. [49] reported that rs1974226 AA genotype and GG/GA vs. AA model were associated with a 2.56 and 2.60-fold increased risk of gastric cancer in the Chinese population, respectively. Additionally, Nakada et al. [50] reported that the rs1974226 GG genotype was associated with a 2.19-fold increased risk of Gram-positive infection compared with GA/AA genotype, and the rs1974226 G allele was associated with a 1.44 and 1.67-fold increased 28 day mortality in St Paul’s Hospital and Vasopressin and Septic Shock Trial cohorts, respectively. However, we didn’t find any association between rs1974226 polymorphism and IS risk in the Chinese population in our present study.

Several possibilities should be taken into account to explain the controversial between IL-17A polymorphisms and susceptibility to different types of human diseases. As for the inconsistent between IL-17A polymorphisms and risk of different human diseases in diverse cohorts of population, it has been well accepted that genetic polymorphisms usually have distinct effect in different types of human diseases, especially in diverse ethnic groups. This phenomenon, such as, can be seen in previous study of genetic association of CD40 gene rs1883832 polymorphism and susceptibility to different types of human cardiovascular diseases, in which association studies of the rs1883832 polymorphism and CAD supported the viewpoint that the rs1883832 CT and CC genotypes were associated with increased risk of CAD [51, 52], however, on the contrary, studies on the association between rs1883832 and IS have demonstrated that the rs1883832 CT and TT genotypes were associated with increased risk of IS [53, 54]. As is shown in the Table 4, we found that the genotype distribution of rs2275913 polymorphism was significantly different from HM-JPT, HM-LWK, HM-MEX, HM-GIH, HM-YRI, HM-ASW, HM-MKK HM-CEU and HM-TSI populations, but were no significantly different when comparing with three other Chinese population (HM-HCB, HM-CHB, and HM-CHD). As for the controversial between IL-17A po1ymorphisms and risk of the same disease, a relative small sample size and the possible selection bias may be potential problems.

IL-17A, produced mainly by activated Th17 cells, can induce the secretion of a variety of stroke-related cytokines, such as IL-6, TNF-α, IL-1β, CXCL1 and CXCL8 [19–24]. The IL-17A levels were significantly increased in serums, cells surface, and atherosclerotic plaques of patients with CAD and IS [12, 17, 18]. Atherosclerosis is known as a major cause of IS. Enhanced Th17-cells were found to associate with atherosclerotic lesion formation in IL-18 deficient apolipoprotein E-knockout fed high-fat diet mice [15]. While, blockade of IL-17A in apolipoprotein E-knockout mice can reduce atherosclerotic lesion development, decreases plaque vulnerability, cellular infiltration, and tissue activation [9, 16]. Given the crucial role of IL-17A in IS pathogenesis, the positive results of our present study were biologically reasonable.

Although the results of our present study are promising, several limitations remain to be consideration. Firstly, the relatively small sample size of the present study may result in insufficient statistical power to detect the relationship between IL-17A polymorphisms and IS risk. Secondly, because our research was designed as a hospital based study, we cannot rule out the possibility of selection bias, and the results we got may not be a good representative of general public. Finally, the subjects of our study are all Chinese, and the results we obtained may not be able to directly expand to other ethnic group.

In conclusion, in this study, we report for the first time that the rs2275913 polymorphism in the promoter of IL-17A gene was associated with a significantly increased risk of IS, probably by upregulating the expression of IL-17A cytokine. Further investigations with a larger sample size, especially in different ethnic groups are needed to confirm our results.

## MATERIALS AND METHODS

### Study population

This case-control study was approved by the ethical committee of Affiliated Hospital of Youjiang Medical University for Nationalities. Informed consents were obtained from all the participants. The case group include 392 IS patients who were consecutively recruited from the department of Neurology, Affiliated Hospital of Youjiang Medical University for Nationalities from January 2013 to September 2016. There were 256 male and 136 female patients with a mean age of 61.8 ± 10.1 years in the IS group. All the IS patients were diagnosed on the basis of clinical symptoms and cranial computed tomography and/or magnetic resonance imaging scans. Patients with hemorrhagic, cardiogenic, tumorous, and drugs induced stroke, and those whom with chronic inflammatory and autoimmune diseases were excluded from this study. Moreover, 443 age and gender matched controls who came to the same hospital for routine medical checkup during the same period were recruited. Individuals with cerebrovascular diseases, hereditary diseases, liver diseases, tumors, and informatory or autoimmune diseases were excluded from this study. There were 278 male and 165 female with a mean age of 60.7 ± 10.3 years in the control group. Clinical information such as age, gender, diabetes, hypertension, triglyceride (TG), low-density lipoprotein cholesterol (LDL-C), high-density lipoprotein cholesterol (HDL-C) and fasting serum levels of total cholesterol (TCH) were obtained from medical record review of our hospital. All participants were unrelated Han Chinese from the same geographic region.

### DNA isolation and genotyping

Genomic DNA was isolated from peripheral blood by using a commercial extraction kit (QIANGEN, China). Genotyping methods have been described in our previous work [55]. Briefly, the genotypes of the rs1974226, rs3748067 and rs2275913 polymorphisms were detected by Snapshot SNP genotyping assay. Genotyping results were analyzed by using GeneMapper4.1 (Applied Biosystems). The results were read by two independent research assistants with a blindness of cases and controls. Furthermore, DNA sequencing method was used to confirm our results, and the results were 100% concordant.

### Serum IL-17A determination

Serum samples from IS patients and the control subjects were separated from venous blood at room temperature and stored at -50 °C until use. Serum IL-17A levels of the IS patients and the control subjects were analyzed by an ELISA kits (eBioscience, Austria) strictly in accordance with the protocol of the manufacturer. Developed color reaction was read by a microplate reader (RT-6000, China). The concentration of serum IL-17A was computed by using standard curve constructed with the kit’s standards over the range of 0-200 pg/ml.

### Statistical analysis

All statistical analyses were carried out by using SPSS statistical software package (SPSS Inc., Chicago, IL, USA; version 17.0). Clinical data such as age, TG, LDL-C, HDL-C and TCH are continuous variables and were displayed as mean± SD. If these data were normally distributed, Student’s *t*-test was used, and otherwise, rank-sum test was used. Other data such as gender, diabetes and hypertension are categorical variables and were expressed as proportions. The comparison of these data chi-squared test were used. Hardy-Weinberg equilibrium (HWE) was calculated by chi-squared test. The association between the three polymorphisms and risk of IS were calculated by logistic regression and were evaluated by odds ratio (OR) and 95% confidence interval (CI). ORs and *P* values of the three polymorphisms were adjusted based on age, gender, hypertension, diabetes, TG, LDL-C, HDL-C, and TCH. Haplotype analysis was performed by online SHEsis software [56]. A *P* value less than 0.05 was considered as statistical significant.

## Abbreviations

IL-17A: interleukin-17A
IS: ischemic stroke
ELISA: enzyme-linked immunosorbent assay
CAD: coronary artery disease
TNF-α: tumor necrosis factor-α
CXCL1: chemokine (C-X-C motif) ligand 1
UTR: untranslated region
LD: linkage disequilibrium
TG: hypertension, triglyceride
LDL-C: low-density lipoprotein cholesterol
HDL-C: high-density lipoprotein cholesterol
TCH: fasting serum levels of total cholesterol
OR: odds ratio
CI: confidence interval

## References

[1] Barker-Collo S, Bennett DA, Krishnamurthi RV, Parmar P, Feigin VL, Naghavi M, Forouzanfar MH, Johnson CO, Nguyen G, Mensah GA, Vos T, Murray CJ, Roth GA (2015). Sex differences in stroke incidence, prevalence, mortality and disability-adjusted life years: results from the Global Burden of Disease Study 2013. Neuroepidemiology.

[2] Roger VL, Go AS, Lloyd-Jones DM, Benjamin EJ, Berry JD, Borden WB, Bravata DM, Dai S, Ford ES, Fox CS, Fullerton HJ, Gillespie C, Hailpern SM (2012). Executive summary: heart disease and stroke statistics--2012 update: a report from the American Heart Association. Circulation.

[3] Goljar N, Burger H, Vidmar G, Leonardi M, Marincek C (2011). Measuring patterns of disability using the International Classification of Functioning, Disability and Health in the post-acute stroke rehabilitation setting. J Rehab Med.

[4] Liu L, Wang D, Wong KS, Wang Y (2011). Stroke and stroke care in China: huge burden, significant workload, and a national priority. Stroke.

[5] Donnan GA, Fisher M, Macleod M, Davis SM (2008). Stroke. Lancet.

[6] Brass LM, Isaacsohn JL, Merikangas KR, Robinette CD (1992). A study of twins and stroke. Stroke.

[7] MacClellan LR, Mitchell BD, Cole JW, Wozniak MA, Stern BJ, Giles WH, Brown DW, Sparks MJ, Kittner SJ (2006). Familial aggregation of ischemic stroke in young women: the Stroke Prevention in Young Women Study. Genet Epidemiol.

[8] Dichgans M (2007). Genetics of ischaemic stroke. Lancet Neurol.

[9] Erbel C, Chen L, Bea F, Wangler S, Celik S, Lasitschka F, Wang Y, Bockler D, Katus HA, Dengler TJ (2009). Inhibition of IL-17A attenuates atherosclerotic lesion development in apoE-deficient mice. J Immunol.

[10] Song L, Schindler C (2004). IL-6 and the acute phase response in murine atherosclerosis. Atherosclerosis.

[11] Eid RE, Rao DA, Zhou J, Lo SF, Ranjbaran H, Gallo A, Sokol SI, Pfau S, Pober JS, Tellides G (2009). Interleukin-17 and interferon-gamma are produced concomitantly by human coronary artery-infiltrating T cells and act synergistically on vascular smooth muscle cells. Circulation.

[12] Cheng X, Yu X, Ding YJ, Fu QQ, Xie JJ, Tang TT, Yao R, Chen Y, Liao YH (2008). The Th17/Treg imbalance in patients with acute coronary syndrome. Clin Immunol.

[13] Xie JJ, Wang J, Tang TT, Chen J, Gao XL, Yuan J, Zhou ZH, Liao MY, Yao R, Yu X, Wang D, Cheng Y, Liao YH, Cheng X (2010). The Th17/Treg functional imbalance during atherogenesis in ApoE(-/-) mice. Cytokine.

[14] de Boer OJ, van der Meer JJ, Teeling P, van der Loos CM, Idu MM, van Maldegem F, Aten J, van der Wal AC (2010). Differential expression of interleukin-17 family cytokines in intact and complicated human atherosclerotic plaques. J Pathol.

[15] Pejnovic N, Vratimos A, Lee SH, Popadic D, Takeda K, Akira S, Chan WL (2009). Increased atherosclerotic lesions and Th17 in interleukin-18 deficient apolipoprotein E-knockout mice fed high-fat diet. Mol Immunol.

[16] Smith E, Prasad KM, Butcher M, Dobrian A, Kolls JK, Ley K, Galkina E (2010). Blockade of interleukin-17A results in reduced atherosclerosis in apolipoprotein E-deficient mice. Circulation.

[17] Kostulas N, Pelidou SH, Kivisakk P, Kostulas V, Link H (1999). Increased IL-1beta, IL-8, and IL-17 mRNA expression in blood mononuclear cells observed in a prospective ischemic stroke study. Stroke.

[18] Hashmi S, Zeng QT (2006). Role of interleukin-17 and interleukin-17-induced cytokines interleukin-6 and interleukin-8 in unstable coronary artery disease. Coron Artery Dis.

[19] Kawaguchi M, Kokubu F, Kuga H, Matsukura S, Hoshino H, Ieki K, Imai T, Adachi M, Huang SK (2001). Modulation of bronchial epithelial cells by IL-17. J Allergy Clin Immunol.

[20] Molet S, Hamid Q, Davoine F, Nutku E, Taha R, Page N, Olivenstein R, Elias J, Chakir J (2001). IL-17 is increased in asthmatic airways and induces human bronchial fibroblasts to produce cytokines. J Allergy Clin Immunol.

[21] Jovanovic DV, Di Battista JA, Martel-Pelletier J, Jolicoeur FC, He Y, Zhang M, Mineau F, Pelletier JP (1998). IL-17 stimulates the production and expression of proinflammatory cytokines, IL-beta and TNF-alpha, by human macrophages. J Immunol.

[22] Laan M, Cui ZH, Hoshino H, Lotvall J, Sjostrand M, Gruenert DC, Skoogh BE, Linden A (1999). Neutrophil recruitment by human IL-17 via C-X-C chemokine release in the airways. J Immunol.

[23] Prause O, Laan M, Lotvall J, Linden A (2003). Pharmacological modulation of interleukin-17-induced GCP-2-, GRO-alpha- and interleukin-8 release in human bronchial epithelial cells. Eur J Pharmacol.

[24] Jones CE, Chan K (2002). Interleukin-17 stimulates the expression of interleukin-8, growth-related oncogene-alpha, and granulocyte-colony-stimulating factor by human airway epithelial cells. Am J Respir Cell Mol Biol.

[25] Kwan J, Horsfield G, Bryant T, Gawne-Cain M, Durward G, Byrne CD, Englyst NA (2013). IL-6 is a predictive biomarker for stroke associated infection and future mortality in the elderly after an ischemic stroke. Exp Gerontol.

[26] Waje-Andreassen U, Krakenes J, Ulvestad E, Thomassen L, Myhr KM, Aarseth J, Vedeler CA (2005). IL-6: an early marker for outcome in acute ischemic stroke. Acta Neurol Scand.

[27] Domac FM, Somay G, Misirli H, Erenoglu NY (2007). Tumor necrosis factor alpha serum levels and inflammatory response in acute ischemic stroke. Neurosciences (Riyadh).

[28] Bokhari FA, Shakoori TA, Butt A, Ghafoor F (2014). TNF-alpha: a risk factor for ischemic stroke. J Ayub Med Coll Abbottabad.

[29] Ovanesyan IG, Ovanesyan RA (2016). [Relationship between platelet aggregation and interleukins IL-1beta and IL-4 in the acute stage of ischemic stroke]. [Article in Russian]. Zh Nevrol Psikhiatr Im S S Korsakova.

[30] Ormstad H, Aass HC, Lund-Sorensen N, Amthor KF, Sandvik L (2011). Serum levels of cytokines and C-reactive protein in acute ischemic stroke patients, and their relationship to stroke lateralization, type, and infarct volume. J Neurol.

[31] Losy J, Zaremba J, Skrobanski P (2005). CXCL1 (GRO-alpha) chemokine in acute ischaemic stroke patients. Folia Neuropathol.

[32] Zhao WM, Shayimu P, Liu L, Fang F, Huang XL (2016). Association between IL-17A and IL-17F gene polymorphisms and risk of gastric cancer in a Chinese population. Genet Mol Res.

[33] Long ZW, Yu HM, Wang YN, Liu D, Chen YZ, Zhao YX, Bai L (2015). Association of IL-17 polymorphisms with gastric cancer risk in Asian populations. World J Gastroenterol.

[34] Wang N, Yang J, Lu J, Qiao Q, Bao G, Wu T, He X (2014). IL-17 gene polymorphism is associated with susceptibility to gastric cancer. Tumour Biol.

[35] Xu BL, Li YT, Dong SX, Qi J, Feng HM, Zi L, Yang DY (2016). IL-17 rs2275913 genetic variation contributes to the development of gastric cancer in a Chinese population. Genet Mol Res.

[36] Ocejo-Vinyals JG, de Mateo EP, Hoz MA, Arroyo JL, Aguero R, Ausin F, Farinas MC (2013). The IL-17 G-152A single nucleotide polymorphism is associated with pulmonary tuberculosis in northern Spain. Cytokine.

[37] Du J, Han JC, Zhang YJ, Qi GB, Li HB, Zhang YJ, Cai S (2016). Single-nucleotide polymorphisms of IL-17 gene are associated with asthma susceptibility in an Asian population. Med Sci Monit.

[38] Chen J, Deng Y, Zhao J, Luo Z, Peng W, Yang J, Ren L, Wang L, Fu Z, Yang X, Liu E (2010). The polymorphism of IL-17 G-152A was associated with childhood asthma and bacterial colonization of the hypopharynx in bronchiolitis. J Clin Immunol.

[39] Maalmi H, Beraies A, Charad R, Ammar J, Hamzaoui K, Hamzaoui A (2014). IL-17A and IL-17F genes variants and susceptibility to childhood asthma in Tunisia. J Asthma.

[40] Han L, Lee HS, Yoon JH, Choi WS, Park YG, Nam SW, Lee JY, Park WS (2014). Association of IL-17A and IL-17F single nucleotide polymorphisms with susceptibility to osteoarthritis in a Korean population. Gene.

[41] Shen L, Zhang H, Yan T, Zhou G, Liu R (2015). Association between interleukin 17A polymorphisms and susceptibility to rheumatoid arthritis in a Chinese population. Gene.

[42] Geng GY, Liu HL, Zhao YJ, Wu L, Mao L, Ba N (2015). Correlation between polymorphisms in the IL-17A and IL-17F genes and development of coronary artery disease. Genet Mol Res.

[43] Shuang L, Li Z, Chen F, Cui X, Ning Y, Su Y, Dong M (2015). Association between interleukin-17 gene polymorphisms and risk of coronary artery disease. Int J Clin Exp Pathol.

[44] Zheng XS, Wang S, Ni M (2016). Association between interleukin 17A gene polymorphisms and risk of coronary artery disease. Genet Mol Res.

[45] Su GB, Guo XL, Liu XC, Cui QT, Zhou CY (2016). Association between interleukin-17A polymorphism and coronary artery disease susceptibility in the Chinese Han population. Genet Mol Res.

[46] Vargas-Alarcon G, Angeles-Martinez J, Villarreal-Molina T, Alvarez-Leon E, Posadas-Sanchez R, Cardoso-Saldana G, Ramirez-Bello J, Perez-Hernandez N, Juarez-Rojas JG, Rodriguez-Perez JM, Fragoso JM, Posadas-Romero C (2015). Interleukin-17A gene haplotypes are associated with risk of premature coronary artery disease in Mexican patients from the Genetics of Atherosclerotic Disease (GEA) study. PLoS One.

[47] Li N, Zhu Q, Li Z, Han Q, Zhang G, Chen J, Lv Y, Xing F, Chen Y, Zeng X, Liu Z (2014). IL17A gene polymorphisms, serum IL-17A and IgE levels, and hepatocellular carcinoma risk in patients with chronic hepatitis B virus infection. Mol Carcinog.

[48] Korytina GF, Akhmadishina LZ, Kochetova OV, Aznabaeva YG, Zagidullin Sh Z, Victorova TV (2016). Inflammatory and immune response genes polymorphisms are associated with susceptibility to chronic obstructive pulmonary disease in tatars population from Russia. Biochem Genet.

[49] Zhou F, Qiu LX, Cheng L, Wang MY, Li J, Sun MH, Yang YJ, Wang JC, Jin L, Wang YN, Wei QY (2016). Associations of genotypes and haplotypes of IL-17 with risk of gastric cancer in an eastern Chinese population. Oncotarget.

[50] Nakada TA, Russell JA, Boyd JH, Walley KR (2011). IL17A genetic variation is associated with altered susceptibility to Gram-positive infection and mortality of severe sepsis. Crit Care.

[51] Wang M, Li Y, Li W, Xia ZE, Wu Q (2011). The CD40 gene polymorphism rs1883832 is associated with risk of acute coronary syndrome in a Chinese case-control study. DNA Cell Biol.

[52] Yan J, Wang C, Du R, Liu P, Chen G (2010). Association analysis of CD40 gene polymorphism with acute coronary syndrome. Clin Exp Med.

[53] Ma Y, Wang SX, Liu Y, Peng GG, Wang XM, Zhang B, Wu BH, Yu JM (2013). Single nucleotide polymorphism of CD40 in the 5’-untranslated region is associated with ischemic stroke. Gene.

[54] Zhang B, Wu T, Song C, Chen M, Li H, Guo R (2013). Association of CD40--1C/T polymorphism with cerebral infarction susceptibility and its effect on sCD40L in Chinese population. Int Immunopharmacol.

[55] Guo J, Xiang Y, Peng YF, Huang HT, Lan Y, Wei YS (2016). The association of novel IL-33 polymorphisms with sIL-33 and risk of systemic lupus erythematosus. Mol Immunol.

[56] Shi YY, He L (2005). SHEsis, a powerful software platform for analyses of linkage disequilibrium, haplotype construction, and genetic association at polymorphism loci. Cell Res.

